# Magnon magic angles and tunable Hall conductivity in 2D twisted ferromagnetic bilayers

**DOI:** 10.1038/s41598-020-72000-y

**Published:** 2020-09-15

**Authors:** Doried Ghader

**Affiliations:** College of Engineering and Technology, American University of the Middle East, Eqaila, Kuwait

**Keywords:** Two-dimensional materials, Topological insulators, Magnetic properties and materials

## Abstract

Twistronics is currently one of the most active research fields in condensed matter physics, following the discovery of correlated insulating and superconducting phases in twisted bilayer graphene (tBLG). Here, we present a magnonic analogue of tBLG. We study magnons in twisted ferromagnetic bilayers (tFBL) with collinear magnetic order, including exchange and weak Dzyaloshinskii-Moriya interactions (DMI). For negligible DMI, tFBL presents discrete magnon magic angles and flat moiré minibands analogous to tBLG. The DMI, however, changes the picture and renders the system much more exotic. The DMI in tFBL induces a rich topological magnon band structure for any twist angle. The twist angle turns to a control knob for the magnon valley Hall and Nernst conductivities. Gapped flat bands appear in a continuum of magic angles in tFBL with DMI. In the lower limit of the continuum, the band structure reconstructs to form several topological flat bands. The luxury of twist-angle control over band gaps, topological properties, number of flat bands, and valley Hall and Nernst conductivities renders tFBL a novel device from fundamental and applied perspectives.

## Introduction

Two-dimensional (2D) materials with intrinsic magnetism has recently been realized^1,2^, opening new horizons in 2D material research^3–22^. In these bosonic Dirac materials, magnetic anisotropy is found to overcome thermal fluctuations and stabilize the magnetic order at finite temperatures. The exotic physics in 2D magnetic systems attracted important attention in search for novel nanomagnetic quantum devices.

To a large extent, the theoretical investigation and experimental realization of bosonic Dirac materials was motivated by their fermionic counterparts. Research on graphene demonstrates that the electronic properties in bilayers change drastically compared to single layer graphene^23,24^. A particularly interesting class of bilayer graphene is the twisted bilayer graphene (tBLG), presenting moiré Bloch bands as a result of the twist. tBLG was found to present fascinating electronic and optical properties, giving rise to novel physics that is completely absent in AB stacked bilayer graphene^26–33^. The twist angle reconstructs the electronic structure, realizing flat moiré superlattice minibands at discrete magic angles. Superconductivity was observed at magic angles in tBLG^32,33^ which triggered unprecedented interest in 2D moiré materials^34–40^. Numerous fermionic 2D heterostructure are currently under intensive investigation for superconducting, correlation and topological features.

Magnons in 2D magnetic materials mimic electrons in 2D fermionic systems^13^. For example, the exchange magnon spectrum in a 2D honeycomb ferromagnet is qualitatively identical to the electronic structure in graphene. Moreover, 2D and quasi-2D quantum magnets with Dzyaloshinskii-Moriya (DM) spin–orbit interaction can host topological magnon bands^5,9,12,16^, similar to their fermionic counterparts. The topological nature of the magnon spectrum in 2D magnets can be confirmed experimentally via measurements on the thermal magnon Hall response. This gives value for the theoretical investigation of magnon Hall conductivity, widely explore in honeycomb ferromagnets with DMI^6,7^.

Given the remarkable analogy between graphene and honeycomb ferromagnets, it is reasonable to propose tFBL with ferromagnetic interlayer coupling (e.g. $CrBr_{3}$ and $Cr_{2} Ge_{2} Te_{6}$) as potential magnonic analogues for tBLG. A wide spectrum of layered 2D magnetic materials are van der Waals materials with a weak interlayer exchange coupling compared to the intralayer exchange^7,9,15,16^. The ferromagnetic interlayer exchange thus mimics the interlayer hopping in tBLG and the arguments in the Bistritzer—MacDonald approach^28^ can hence be implemented to develop the tFBL spin wave theory. We find that the magnons transport properties in tFBL indeed mimic their electronic counterparts. The DMI, however, enriches the topology in the system and induces exciting new physics. Unlike tBLG, its magnetic twin with DMI presents a continuum of magic angles and numerous topological flat bands. The magnon bands valley Berry curvatures and valley Chern numbers are sensitive to the twist angle and the DMI strength. The valley thermal magnon Hall and Nernst conductivities induced by the multiple topological flat bands show a complex and exotic response to the twist angle. The twist angle can hence be used as a control knob for these topological responses, which is not possible in tBLG.

## Results

### Magnons in a ferromagnetic monolayer

**Figure 1:**
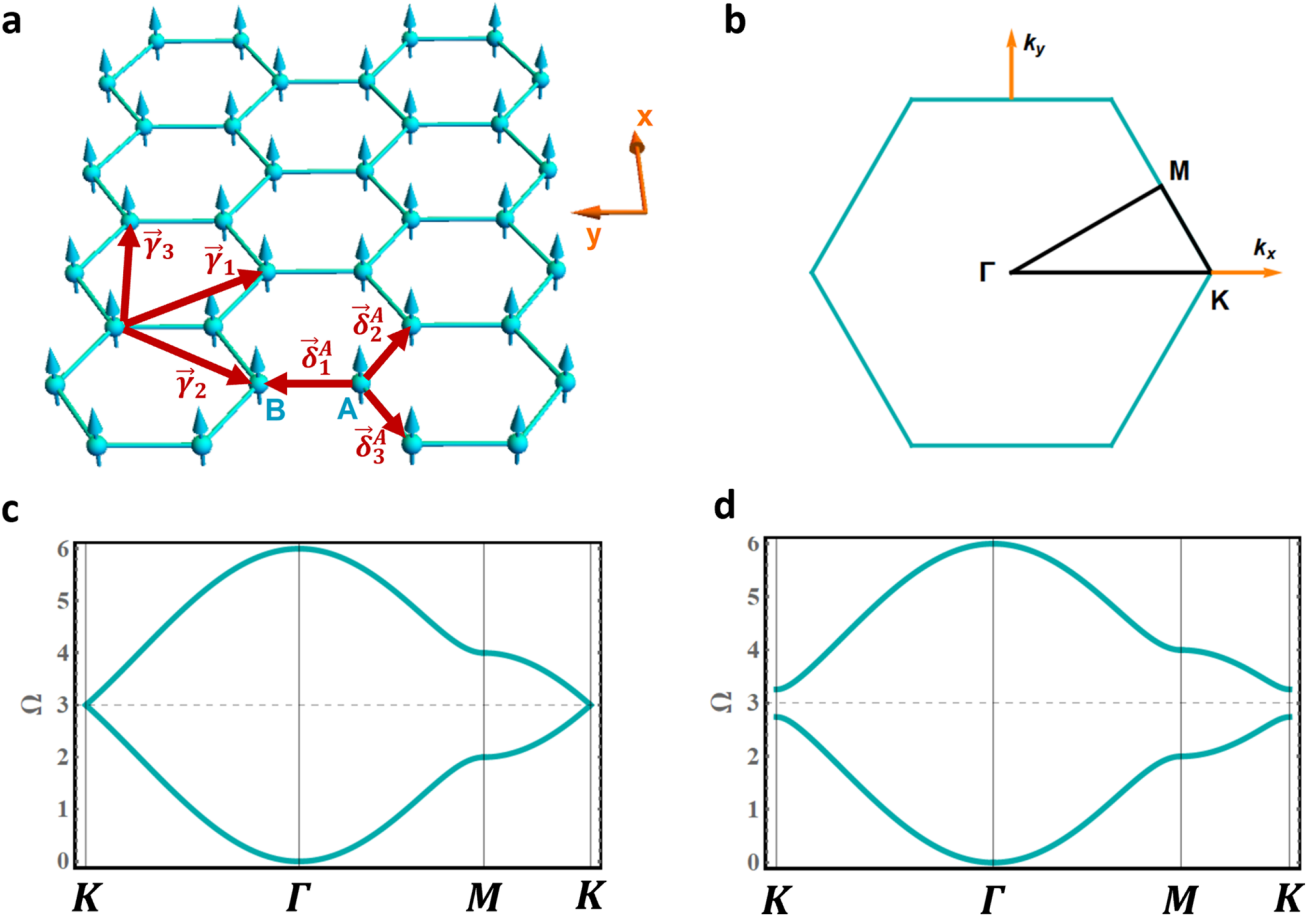
(**a**) Schematic representation of a single ferromagnetic honeycomb sheet. (**b**) The corresponding Brillouin zone and high symmetry axes. (**c**) and (**d**) show the magnon dispersion curves along the high symmetry axes for $D = 0$ and $D = 0.05J$ respectively. Figures generated using Mathematica Software version 12 (free trial) https://www.wolfram.com/mathematica/.

We start with a ferromagnetic honeycomb monolayer (Fig. 1a) with nearest neighbor exchange and next nearest neighbors DMI. We define the lattice constant $a$ as the $A - A$ (or $B - B$) distance, whereas the nearest-neighbor distance is denoted $d = a/\sqrt 3$. The vectors connecting an A-site to its three nearest neighbors can be expressed as $\vec{\delta }_{1}^{A} = a\left( {0, 1/\sqrt 3 } \right)$, $\vec{\delta }_{2}^{A} = a\left( {1/2, - \sqrt 3 /6} \right)$, and $\vec{\delta }_{3}^{A} = a\left( { - 1/2, - \sqrt 3 /6} \right)$. For the DMI, the next nearest neighbors vectors for both A and B sublattices read $\vec{\gamma }_{1} { } = a\left( {1/2, - \sqrt 3 /2} \right)$, $\vec{\gamma }_{2} { } = a\left( { - 1/2, - \sqrt 3 /2} \right)$, $\vec{\gamma }_{3} { } = a\left( {1, 0} \right)$, $\vec{\gamma }_{4} = - \vec{\gamma }_{1}$, $\vec{\gamma }_{5} = - \vec{\gamma }_{2}$, and $\vec{\gamma }_{6} = - \vec{\gamma }_{3}$. A schematic illustration of vectors $\vec{\delta }_{i}^{A}$ and $\vec{\gamma }_{j}$ is presented in Fig. 1a.

We will use $J$ and $D$ to denote the exchange and DMI coefficients respectively. The real space semi-classical Heisenberg Hamiltonian for the ferromagnetic monolayer (ML) can be expressed as

$$\begin{aligned} {\mathcal{H}}_{ML} & = - J\mathop \sum \limits_{{\vec{R}_{A} ,\vec{\delta }_{i}^{A} }} \vec{S}^{A} \left( {\vec{R}_{A} ,t} \right).\vec{S}^{B} \left( {\vec{R}_{A} + \vec{\delta }_{i}^{A} ,t} \right) \\ & \quad + \mathop \sum \limits_{{\vec{R}_{A} ,\vec{\gamma }_{j} }} \vec{D}\left( {\vec{R}_{A} ,\vec{R}_{A} + \vec{\gamma }_{j} } \right).\left[ { \vec{S}^{A} \left( {\vec{R}_{A} ,t} \right) \times \vec{S}^{A} \left( {\vec{R}_{A} + \vec{\gamma }_{j} ,t} \right)} \right] \\ & \quad + \mathop \sum \limits_{{\vec{R}_{B} ,\vec{\gamma }_{j} }} \vec{D}\left( {\vec{R}_{B} ,\vec{R}_{B} + \vec{\gamma }_{j} } \right).\left[ {\vec{S}^{B} \left( {\vec{R}_{B} ,t} \right) \times \vec{S}^{B} \left( {\vec{R}_{B} + \vec{\gamma }_{j} ,t} \right)} \right] \\ \end{aligned}$$

Here, $\vec{S}^{\alpha } \left( {\vec{R}_{\alpha } ,t} \right)$ is the spin at site $\vec{R}_{\alpha }$ and time $t$. The alternating DMI vector has the form $\vec{D}\left( {\vec{r},\vec{r} + \vec{\gamma }_{j} } \right) = D_{z} \hat{z} = \pm D\hat{z}$, where the orientation of $\vec{D}$ is determined in the conventional way from the local geometry of the honeycomb lattice ^5^.

In the semi-classical approach, $\vec{S}^{\alpha }$ is treated as a numerical vector and the spin dynamics are governed by the Landau-Lifshitz (LL) equations of motion. In Supplementary Notes 1, we use this approach to derive the monolayer momentum-space Hamiltonian. We arrive at

$${\mathcal{H}}_{ML} \left( {\vec{k}} \right) = JM_{z} \left( {\begin{array}{*{20}c} {3 - \frac{i D}{J}f_{D} \left( {\vec{k}} \right)} & { - f\left( {\vec{k}} \right)} \\ { - f^{*} \left( {\vec{k}} \right)} & {3 + \frac{i D}{J}f_{D} \left( {\vec{k}} \right)} \\ \end{array} } \right)$$

with the exchange and DMI functions,$f\left( {\vec{k}} \right)$ and $f_{D} \left( {\vec{k}} \right)$ respectively, given by

$$f\left( {\vec{k}} \right) = e^{{ik_{y} \frac{a}{\sqrt 3 } }} + 2e^{{ - i\frac{\sqrt 3 a}{6} k_{y} }} \cos \left( {\frac{a}{2}k_{x} } \right)$$

$$f_{D} \left( {\vec{k}} \right) = 4 i\sin\left( {\frac{a}{2}k_{x} } \right)\cos \left( {\frac{\sqrt 3 a}{2} k_{y} } \right) - 2 i \sin\left( {k_{x} a} \right)$$

$M_{z}$ is the constant $z -$ component of the magnetization and $\vec{k} = \left( {k_{x} ,k_{y} } \right)$ denotes the 2D momentum.

Similar to graphene tight-binding Hamiltonian, ${\mathcal{H}}_{ML} \left( {\vec{k}} \right)$ can be expanded near $K$ and $K^{\prime} = - K$ valleys in the form of Dirac Hamiltonians,

$${\mathcal{H}}_{ML}^{K} \left( {\vec{K} + \vec{q}} \right) = 3JM_{z} I_{2} + 3\sqrt 3 DM_{z} \sigma_{z} + v\left| {\vec{q}} \right|\left( {\begin{array}{*{20}c} 0 & {e^{{ - i\theta_{{\vec{q}}} }} } \\ {e^{{i\theta_{{\vec{q}}} }} } & 0 \\ \end{array} } \right)$$

$${\mathcal{H}}_{ML}^{ - K} \left( { - \vec{K} + \vec{q}} \right) = 3JM_{z} I_{2} - 3\sqrt 3 DM_{z} \sigma_{z} - v\left| {\vec{q}} \right|\left( {\begin{array}{*{20}c} 0 & {e^{{i\theta_{{\vec{q}}} }} } \\ {e^{{ - i\theta_{{\vec{q}}} }} } & 0 \\ \end{array} } \right)$$

with $v = \frac{1}{2}\sqrt 3 JM_{z} a$, $\vec{K} = \left( {0,\frac{4\pi }{{3a}}} \right)$, and $\vec{\sigma } = \left( {\sigma_{x} ,\sigma_{y} } \right)$. The parameter $v$ can be interpreted as the magnitude of the magnon group velocity near the valleys. $\pm \vec{K}$ are the momenta vectors from the center of the Brillouin Zone (BZ) to the valleys $\pm K$. $\vec{q}$ represents a small deviation from $\pm \vec{K}$ in the momentum-space. The matrices $\sigma_{i}$ are the Pauli matrices while $I_{2}$ is the $2 \times 2$ identity matrix. $\theta_{{\vec{q}}}$ is the angle between momentum $\vec{q}$ and the $k_{x} -$ axis.

Figure 1b presents the BZ and highlights the high symmetry axes $K\Gamma$, $\Gamma M$, and $MK$. Figure 1c and Fig. 1d present the magnon spectra along the high symmetry axes for $D = 0$ and $D = 0.05J$ respectively. For negligible DMI, the magnons act as massless Dirac quasi-particles near $K$, with linear dispersions. Similar to tBLG, magnon flat bands are expected in tFBL, as a result of the band repulsion effect between the overlapping Dirac cones from different layers. The DMI, however, induces a band gap at the valleys and significantly reduces the valley group velocities (Fig. 1d). The Dirac cones are absent in this case and the band repulsion effect is expected to induce new dispersion profiles that are absent in tBLG.

### Model Hamiltonian for tFBL

**Figure 2:**
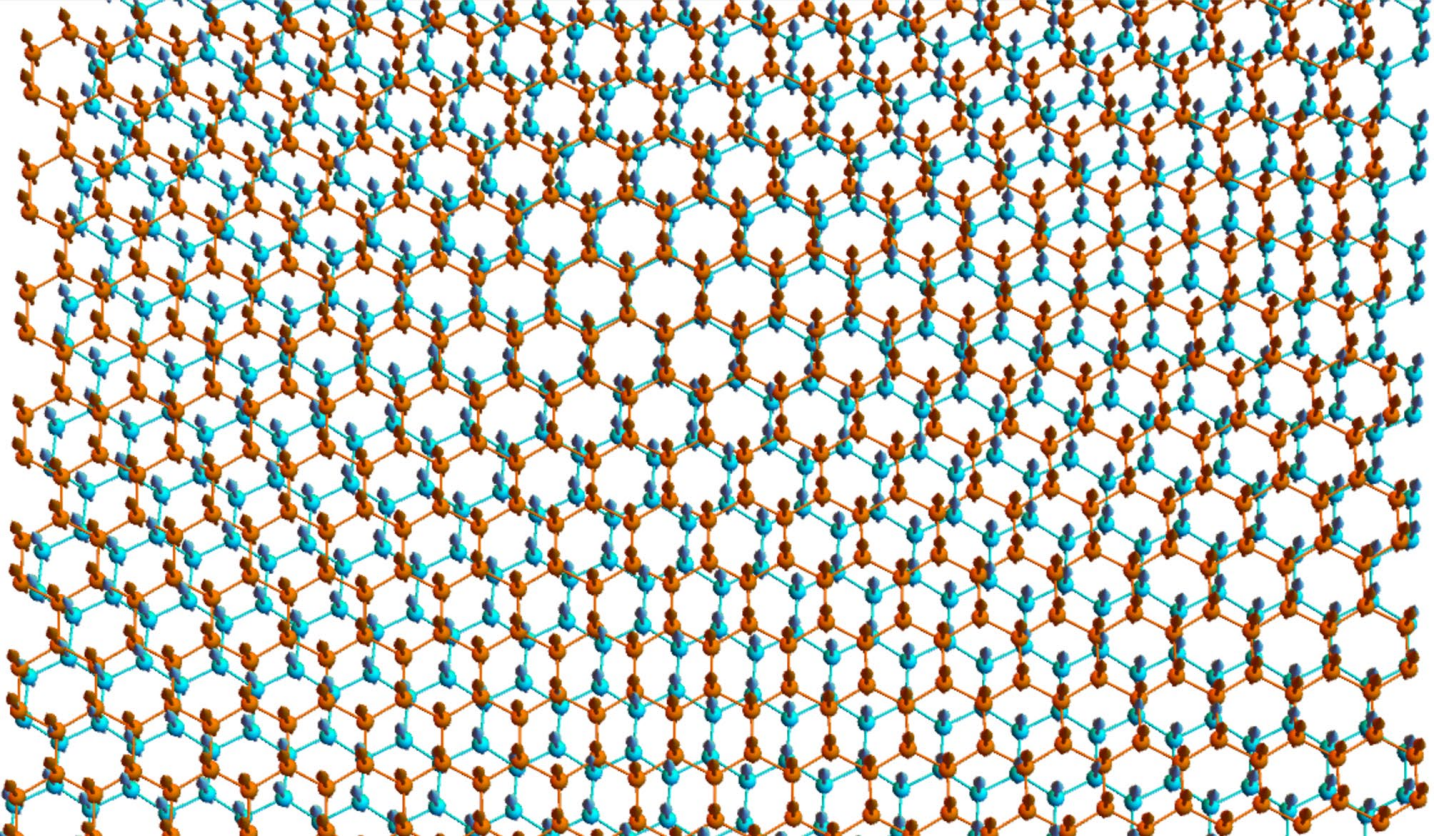
Schematic representation of tFBL ($\theta = 5^\circ$). The figure present a representative part of the quasi-infinite bilayer. Figure generated using Mathematica Software version 12 (free trial) https://www.wolfram.com/mathematica/.

Consider next a ferromagnetic bilayer in the AB configuration. Sites in layers $l = 1, 2$ are denoted $A_{l}$ and $B_{l}$. In the AB stacking, the constant ferromagnetic interlayer exchange coefficient, $J_{ \bot }$, is considered between $A_{1} - B_{2}$ dimers and neglected elsewhere. To form the tFBL, we translate layer 2 by a vector $\vec{\tau }_{0} = \left( {\tau_{0x} ,\tau_{0y} } \right)$ and then rotate layers 1 and 2 in opposite directions. To be specific, layer 1 and 2 are rotated by $\theta /2$ in clockwise and anticlockwise directions respectively. The tFBL is assumed in a collinear ground state, with a sufficiently weak ferromagnetic interlayer exchange and DMI compared to the intralayer ferromagnetic coupling. A schematic representation is presented in Fig. 2.

We write a semi-classical Heisenberg Hamiltonian ${\mathcal{H}}_{T}$ in real space, including nearest neighbor exchange and next nearest neighbors DMI,

1 $$\begin{aligned} {\mathcal{H}}_{T} & = - J\mathop \sum \limits_{{l, \vec{\delta }_{i}^{A} }} \vec{S}^{{A_{l} }} \left( {\vec{R}_{{A_{l} }} ,t} \right).\vec{S}^{{B_{l} }} \left( {\vec{R}_{{A_{l} }} + \vec{\delta }_{i}^{A} ,t} \right) - \mathop \sum \limits_{\alpha ,\beta } J_{ \bot } \left( {\vec{R}_{{\alpha_{1} }} ,\vec{R}_{{\beta_{2} }} } \right) \vec{S}^{{\alpha_{1} }} \left( {\vec{R}_{{\alpha_{1} }} ,t} \right).\vec{S}^{{\beta_{2} }} \left( {\vec{R}_{{\beta_{2} }} ,t} \right) \\ & \quad + \mathop \sum \limits_{{\alpha ,l,\vec{\gamma }_{j} }} \vec{D}\left( {\vec{R}_{{\alpha_{l} }} ,\vec{R}_{{\alpha_{l} }} + \vec{\gamma }_{j} } \right).\left[ { \vec{S}^{{\alpha_{l} }} \left( {\vec{R}_{{\alpha_{l} }} ,t} \right) \times \vec{S}^{{\alpha_{l} }} \left( {\vec{R}_{{\alpha_{l} }} + \vec{\gamma }_{j} ,t} \right)} \right] \\ \end{aligned}$$

Index $l$ is summed over 1 and 2 while each of $\alpha$ and $\beta$ runs over $A$ and $B$ sites. $J_{ \bot } \left( {\vec{R}_{{\alpha_{1} }} ,\vec{R}_{{\beta_{2} }} } \right)$ is the distance dependent interlayer exchange coefficient between spins at sites $\vec{R}_{{\alpha_{1} }}$ and $\vec{R}_{{\beta_{2} }}$. The first, second and third terms in ${\mathcal{H}}_{T}$ hence account for the intralayer exchange, interlayer exchange and DM interactions respectively. A less compact expression for ${\mathcal{H}}_{T}$ is presented in Supplementary Notes 2.

### Spin dynamics in tFBL

The DMI term in ${\mathcal{H}}_{T}$ can be rewritten in terms of a scalar product^22^ which unifies the treatment of the exchange and the DMI parts of ${\mathcal{H}}_{T}$ (Supplementary Notes 1 and 3). The effective exchange fields $\vec{H}^{{\alpha_{l} }}$ acting on the sublattice magnetizations $\vec{M}^{{\alpha_{l} }}$ can then be derived from the Heisenberg Hamiltonian as^19–22,41–46^

2 $$\begin{aligned} \vec{H}^{{\alpha_{l} }} \left( {\vec{R}_{{\alpha_{l} }} ,t} \right) & = - J\mathop \sum \limits_{{\vec{\delta }_{i}^{\alpha } }} \vec{M}^{{\overline{\alpha }_{l} }} \left( {\vec{R}_{{\alpha_{l} }} + \vec{\delta }_{i}^{\alpha } ,t} \right) + \mathop \sum \limits_{{\vec{\gamma }_{j} }} D_{z} \left( {\vec{R}_{{\alpha_{l} }} ,\vec{R}_{{\alpha_{l} }} + \vec{\gamma }_{j} } \right)\vec{M}_{D}^{{\alpha_{l} }} \left( {\vec{R}_{{\alpha_{l} }} + \vec{\gamma }_{j} ,t} \right) \\ & \quad - \mathop \sum \limits_{{\vec{R}_{{\alpha_{{\overline{l}}} }} }} J_{ \bot } \left( {\vec{R}_{{\alpha_{l} }} ,\vec{R}_{{\alpha_{{\overline{l}}} }} } \right) \vec{M}^{{\alpha_{{\overline{l}}} }} \left( {\vec{R}_{{\alpha_{{\overline{l}}} }} ,t} \right) - \mathop \sum \limits_{{\vec{R}_{{\overline{\alpha }_{{\overline{l}}} }} }} J_{ \bot } \left( {\vec{R}_{{\alpha_{l} }} ,\vec{R}_{{\overline{\alpha }_{{\overline{l}}} }} } \right) \vec{M}^{{\overline{\alpha }_{{\overline{l}}} }} \left( {\vec{R}_{{\overline{\alpha }_{{\overline{l}}} }} ,t} \right) \\ \end{aligned}$$

where we have used the convention that if $\alpha = A$ then $\overline{\alpha } = B$ and vice versa. Same convention assumed for $l$ and $\overline{l}$. We also introduce the vector $\vec{M}_{D}^{{\alpha_{l} }} = M_{y}^{{\alpha_{l} }} \hat{x} - M_{x}^{{\alpha_{l} }} \hat{y}$ to simplify the expression of $\vec{H}^{{\alpha_{l} }}$.

In Supplementary notes 3, we present a detailed development of the LL equations of motion, $\partial_{t} \vec{M}^{{\alpha_{l} }} = \vec{M}^{{\alpha_{l} }} \times \vec{H}^{{\alpha_{l} }}$. Interestingly, the interlayer terms in the tFBL are found to be qualitatively identical to those encountered in the electronic theory for tBLG. These are hence treated using the Bistritzer—MacDonald continuum approach^28^, valid for commensurate and incommensurate structures at small twist angles ($\theta \le 10^\circ$). The spin wave theory, however, yields diagonal terms that are absent in the electronic theory. Nevertheless, the main ideas of the Bistritzer—MacDonald approach can still be applied to evaluate these terms (details in Supplementary notes 3). We prove that, unlike AB-stacked ferromagnetic bilayers, the exchange interaction contribution to the diagonal terms in the tFBL Hamiltonian is uniform and only causes a shift in the magnonic spectrum. With the development in Supplementary notes 3, the $K -$ valley LL equations (near $K_{l}$ and $K_{{\overline{l}}}$) reduce to

3a $$\begin{aligned} {\Omega } u_{{A_{1} }} \left( {\vec{K}_{1} + \vec{q}} \right) & = \left[ {{\Omega }_{0} + 3\sqrt 3 \frac{D}{J}} \right]u_{{A_{1} }} \left( {\vec{K}_{1} + \vec{q}} \right) + \frac{\sqrt 3 a}{2}\left| {\vec{q}} \right|e^{{ - i\left( {\theta_{q} - \theta /2} \right)}} u_{{B_{1} }} \left( {\vec{K}_{1} + \vec{q}} \right) \\ & \quad - \frac{{J_{ \bot } }}{3J}\left[ {u_{{A_{2} }} \left( {\vec{K}_{2} + \vec{q} + \vec{q}_{b} } \right) + e^{i\varphi } u_{{A_{2} }} \left( {\vec{K}_{2} + \vec{q} + \vec{q}_{Jr} } \right) + e^{ - i\varphi } u_{{A_{2} }} \left( {\vec{K}_{2} + \vec{q} + \vec{q}_{Jl} } \right)} \right] \\ & \quad - \frac{{J_{ \bot } }}{3J}\left[ {u_{{B_{2} }} \left( {\vec{K}_{2} + \vec{q} + \vec{q}_{b} } \right) + u_{{B_{2} }} \left( {\vec{K}_{2} + \vec{q} + \vec{q}_{Jr} } \right) + u_{{B_{2} }} \left( {\vec{K}_{2} + \vec{q} + \vec{q}_{Jl} } \right)} \right] \\ \end{aligned}$$

3b $$\begin{aligned} {\Omega } u_{{B_{1} }} \left( {\vec{K}_{1} + \vec{q}} \right) & = \left[ {{\Omega }_{0} - 3\sqrt 3 \frac{D}{J}} \right]u_{{B_{1} }} \left( {\vec{K}_{1} + \vec{q}} \right) + \frac{\sqrt 3 a}{2}\left| {\vec{q}} \right|e^{{i\left( {\theta_{q} - \theta /2} \right)}} u_{{A_{1} }} \left( {\vec{K}_{1} + \vec{q}} \right) \\ & \quad - \frac{{J_{ \bot } }}{3J}\left[ {u_{{A_{2} }} \left( {\vec{K}_{2} + \vec{q} + \vec{q}_{b} } \right) + e^{ - i\varphi } u_{{A_{2} }} \left( {\vec{K}_{2} + \vec{q} + \vec{q}_{Jr} } \right) + e^{i\varphi } u_{{A_{2} }} \left( {\vec{K}_{2} + \vec{q} + \vec{q}_{Jl} } \right)} \right] \\ & \quad - \frac{{J_{ \bot } }}{3J}\left[ {u_{{B_{2} }} \left( {\vec{K}_{2} + \vec{q} + \vec{q}_{b} } \right) + e^{i\varphi } u_{{B_{2} }} \left( {\vec{K}_{2} + \vec{q} + \vec{q}_{Jr} } \right) + e^{i\varphi } u_{{B_{2} }} \left( {\vec{K}_{2} + \vec{q} + \vec{q}_{Jl} } \right)} \right] \\ \end{aligned}$$

3c $$\begin{aligned} {\Omega } u_{{A_{2} }} \left( {\vec{K}_{2} + \vec{q}} \right) & = \left[ {{\Omega }_{0} + 3\sqrt 3 \frac{D}{J}} \right]u_{{A_{2} }} \left( {\vec{K}_{2} + \vec{q}} \right) + \frac{\sqrt 3 a}{2}\left| {\vec{q}} \right|e^{{ - i\left( {\theta_{q} + \theta /2} \right)}} u_{{B_{2} }} \left( {\vec{K}_{2} + \vec{q}} \right) \\ & \quad - \frac{{J_{ \bot } }}{3J}\left[ {u_{{A_{1} }} \left( {\vec{K}_{1} + \vec{q} + \vec{q}_{b} } \right) + e^{ - i\varphi } u_{{A_{1} }} \left( {\vec{K}_{1} + \vec{q} + \vec{q}_{Jr} } \right) + e^{i\varphi } u_{{A_{1} }} \left( {\vec{K}_{1} + \vec{q} + \vec{q}_{Jl} } \right)} \right] \\ & \quad - \frac{{J_{ \bot } }}{3J}\left[ {u_{{B_{1} }} \left( {\vec{K}_{1} + \vec{q} + \vec{q}_{b} } \right) + e^{i\varphi } u_{{B_{1} }} \left( {\vec{K}_{1} + \vec{q} + \vec{q}_{Jr} } \right) + e^{ - i\varphi } u_{{B_{1} }} \left( {\vec{K}_{1} + \vec{q} + \vec{q}_{Jl} } \right)} \right] \\ \end{aligned}$$

3d $$\begin{aligned} {\Omega } u_{{B_{2} }} \left( {\vec{K}_{2} + \vec{q}} \right) & = \left[ {{\Omega }_{0} - 3\sqrt 3 \frac{D}{J}} \right]u_{{B_{2} }} \left( {\vec{K}_{2} + \vec{q}} \right) + \frac{\sqrt 3 a}{2}\left| {\vec{q}} \right|e^{{i\left( {\theta_{q} + \theta /2} \right)}} u_{{A_{2} }} \left( {\vec{K}_{2} + \vec{q}} \right) \\ & \quad - \frac{{J_{ \bot } }}{3J}\left[ {u_{{A_{1} }} \left( {\vec{K}_{1} + \vec{q} + \vec{q}_{b} } \right) + u_{{A_{1} }} \left( {\vec{K}_{1} + \vec{q} + \vec{q}_{Jr} } \right) + u_{{A_{1} }} \left( {\vec{K}_{1} + \vec{q} + \vec{q}_{Jl} } \right)} \right] \\ & \quad - \frac{{J_{ \bot } }}{3J}\left[ {u_{{B_{1} }} \left( {\vec{K}_{1} + \vec{q} + \vec{q}_{b} } \right) + e^{ - i\varphi } u_{{B_{1} }} \left( {\vec{K}_{1} + \vec{q} + \vec{q}_{Jr} } \right) + e^{i\varphi } u_{{B_{1} }} \left( {\vec{K}_{1} + \vec{q} + \vec{q}_{Jl} } \right)} \right] \\ \end{aligned}$$

In Eq. 3, $u$ denotes the Bloch wave amplitude, ${\Omega }$ is the magnon frequency normalized with respect to $JM_{z}$, and $\varphi = 2\pi /3$. The symbol ${\Omega }_{0} = 3 + \frac{{2\tilde{J}_{ \bot } \left( 0 \right)}}{JA}$ accounts for the uniform exchange interaction contribution (discussed above) to the diagonal terms. $A$ is the honeycomb unit cell area ($A = \sqrt 3 a^{2} /2$) and $\tilde{J}_{ \bot } \left( {\vec{q}} \right)$ represents the Fourier transform of the interlayer exchange. We have also defined the constant momenta

$$\vec{q}_{b} = \frac{{8\pi \sin \left( {\theta /2{ }} \right)}}{3\sqrt 3 d}\left( {0, - 1} \right)$$

$$\vec{q}_{Jr} = \frac{{8\pi \sin \left( {\theta /2{ }} \right)}}{3\sqrt 3 d}\left( {\sqrt 3 /2,1/2} \right)$$

$$\vec{q}_{Jl} = \frac{{8\pi \sin \left( {\theta /2{ }} \right)}}{3\sqrt 3 d}\left( { - \sqrt 3 /2,1/2} \right)$$

Equations 3 determine the momentum-space Hamiltonian, ${\mathcal{H}}_{T}^{K} \left( {\vec{q}} \right)$, near the $K -$ valley. The Hamiltonian ${\mathcal{H}}_{T}^{ - K} \left( {\vec{q}} \right)$ can then be deduced in a straight forward manner. Similar to the tBLG theory, the coupled amplitudes in the LL equations do not form a closed set. It is hence necessary to properly truncate the ensemble of LL equations involved in the formalism. The dimensions of ${\mathcal{H}}_{T}^{ \pm K} \left( {\vec{q}} \right)$ should be sufficiently large, notably for small twist angles, to guarantee convergence of the magnon spectrum. In most of our numerical results, we have used $172 \times 172$ matrices and discretized the moiré BZ to 14,000–15,000 points. These dimensions were occasionally increased to ensure proper convergence of the valley Chern numbers.

### Magnon magic angles and topological bands

We performed intensive numerical investigations on the magnon spectrum within the intervals $0.05J \le J_{ \bot } \le 0.4J$ and $0.02 \le D \le 0.3$. This analysis proves that the main results and conclusions are general and hence independent of the particular values of $J_{ \bot }$ and $D$. We only present representative samples of the generated numerical results, fixing the parameters $J_{ \bot } = 0.2 J$ and ${\Omega }_{0} = 3.8$. Obviously, the specific choice of ${\Omega }_{0}$ is irrelevant, as it only shifts the magnon spectrum.

**Figure 3:**
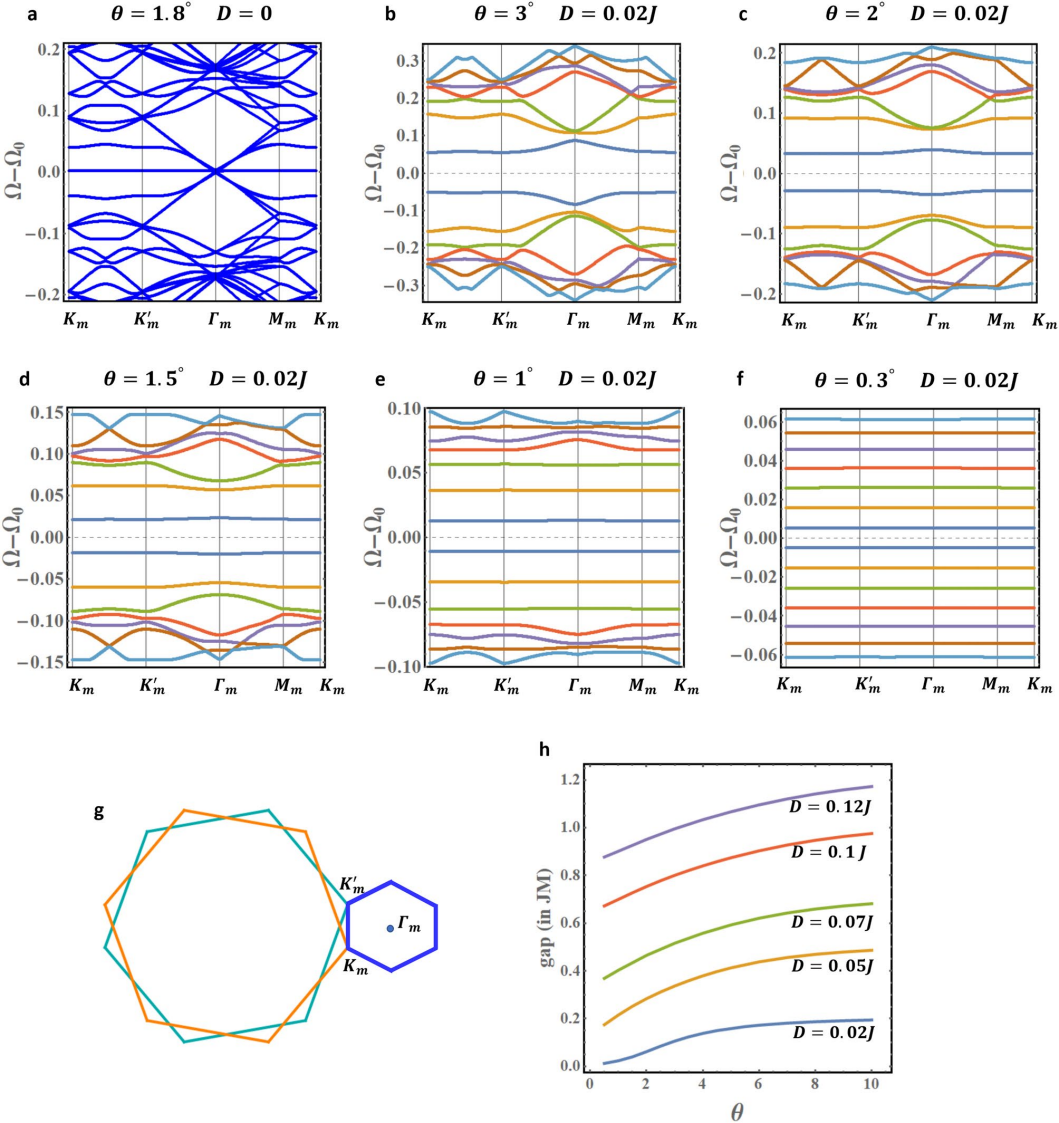
(**a**) Magic angle magnon spectrum for DMI-free tFBL. (**b**–**f**) Reconstruction of the $K -$ valley magnon spectrum for a tFBL with weak DMI ($D = 0.02 J$). At slight twists, the spectrum presents multiple flat bands. (**g**) The rotated Brillouin zones for the 2 layers and the moiré BZ. (**h**) Dependence of the primary gap on the twist angle. Figures generated using Mathematica Software version 12 (free trial) https://www.wolfram.com/mathematica/.

For negligible DMI, the Hamiltonian ${\mathcal{H}}_{T}^{K} \left( {\vec{q}} \right)$ is qualitatively identical to the tBLG Hamiltonian and the magnons in tFBL mimic the electrons in tBLG. The first magic angle for the chosen $J_{ \bot }$ is found at $\theta \approx 1.8^\circ$. The corresponding magnon band structure is presented in Fig. 3a. The spectrum is calculated from both valley contributions and plotted along high symmetry axes in the moiré BZ (Fig. 3g).

Introducing the slightest DMI strongly affects the dispersion profiles. Figure 3b–f illustrate the reconstruction of the $+ K -$ valley magnon bands, caused by the twist angle $\theta$, in tFBL with weak DMI $\left( {D = 0.02 J} \right)$. The figures show the gradual construction of the flat band bundle when $\theta$ is decreased: the bands are squeezed towards lower energies while their bandwidths collapse to very small values compared to the DMI-induced gaps. For clarity, we only present 14 magnon bands near the ${\Omega } = {\Omega }_{0}$ axis. Very similar behavior is observed for the $- K -$ valley magnons (not presented).

Figure 3b–f further demonstrate the continuum of magic angles. For the selected DMI in the figures, (almost) flat bands exist for any angle less than $2^\circ$ (approximately). The upper limit of the continuum increases with the DMI. Another interesting numerical fact is the improved symmetry between positive and negative energies (sign relative to ${\Omega }_{0}$) in the presence of DMI. Additional numerical results on the reconstruction of the magnon spectrum are presented in Supplementary notes 4 for several values of $J_{ \bot }$ and $D$.

To make reference to the different bands simpler, we use the notation $\epsilon_{\mu ,i} \left( {\vec{q}} \right)$ to denote their energies. $\mu$ takes the values $\pm$ in reference to the $\pm K -$ valleys. The bands above ${\Omega }_{0}$ are labeled by $i = 1,{ }2, \ldots$ in ascending energy order, while the bands below ${\Omega }_{0}$ are labeled by $i = - 1,{ } - 2, \ldots$ in descending energy order.

The DMI induces a tunable primary energy gap between the valence-like band, $\epsilon_{ + , - 1}$, and the conduction-like band, $\epsilon_{ + ,1}$. The gap dependence on the twist angle $\theta$ and the DMI strength $D$ is analyzed in Fig. 3h. A consistent behavior is observed for different values of $D$.

The DMI also opens tiny gaps between neighboring bands $\epsilon_{\mu ,i}$ and $\epsilon_{\mu ,i + 1}$. This decouples the bands and enables us to calculate their valley Berry curvatures, ${\Omega }_{\mu ,i}$. For completeness, we present some details on the numerical approach used to calculate ${\Omega }_{\mu ,i}$ following reference ^47^. For a band $\epsilon_{\mu ,i}$ and any $\vec{q}$ in the discretized moiré BZ, we numerically calculate the quantities

$$U_{\mu ,i}^{x} \left( {\vec{q}} \right) = \left\langle {\left. {\epsilon_{\mu ,i} \left( {\vec{q} + \delta q\,\hat{q}_{x} } \right)} \right|\epsilon_{\mu ,i} \left( {\vec{q}} \right)} \right\rangle$$

$$U_{\mu ,i}^{y} \left( {\vec{q}} \right) = \left\langle {\left. {\epsilon_{\mu ,i} \left( {\vec{q} + \delta q\,\hat{q}_{y} } \right)} \right|\epsilon_{\mu ,i} \left( {\vec{q}} \right)} \right\rangle$$

In the above expressions, $\hat{q}_{x}$ and $\hat{q}_{y}$ denote the momentum-space unit vectors while $\delta q$ denotes the infinitesimal segment (or step) used to discretize the moiré BZ. Next, the Wilson loop $W_{\mu ,i} \left( {\vec{q}} \right)$ is calculated from

$$W_{\mu ,i} \left( {\vec{q}} \right) = U_{\mu ,i}^{x} \left( {\vec{q}} \right)U_{\mu ,i}^{y} \left( {\vec{q} + \delta q \hat{q}_{x} } \right)U_{\mu ,i}^{x*} \left( {\vec{q} + \delta q \hat{q}_{y} } \right)U_{\mu ,i}^{y*} \left( {\vec{q}} \right)$$

where $*$ denotes complex conjugation. Finally, ${\Omega }_{\mu ,i}$ can be deduced via the argument ($arg$-function) of the Wilson loop as

$${\Omega }_{\mu ,i} \left( {\vec{q}} \right) = \frac{{\arg W_{\mu ,i} \left( {\vec{q}} \right)}}{{\delta q^{2} }}$$

**Figure 4:**
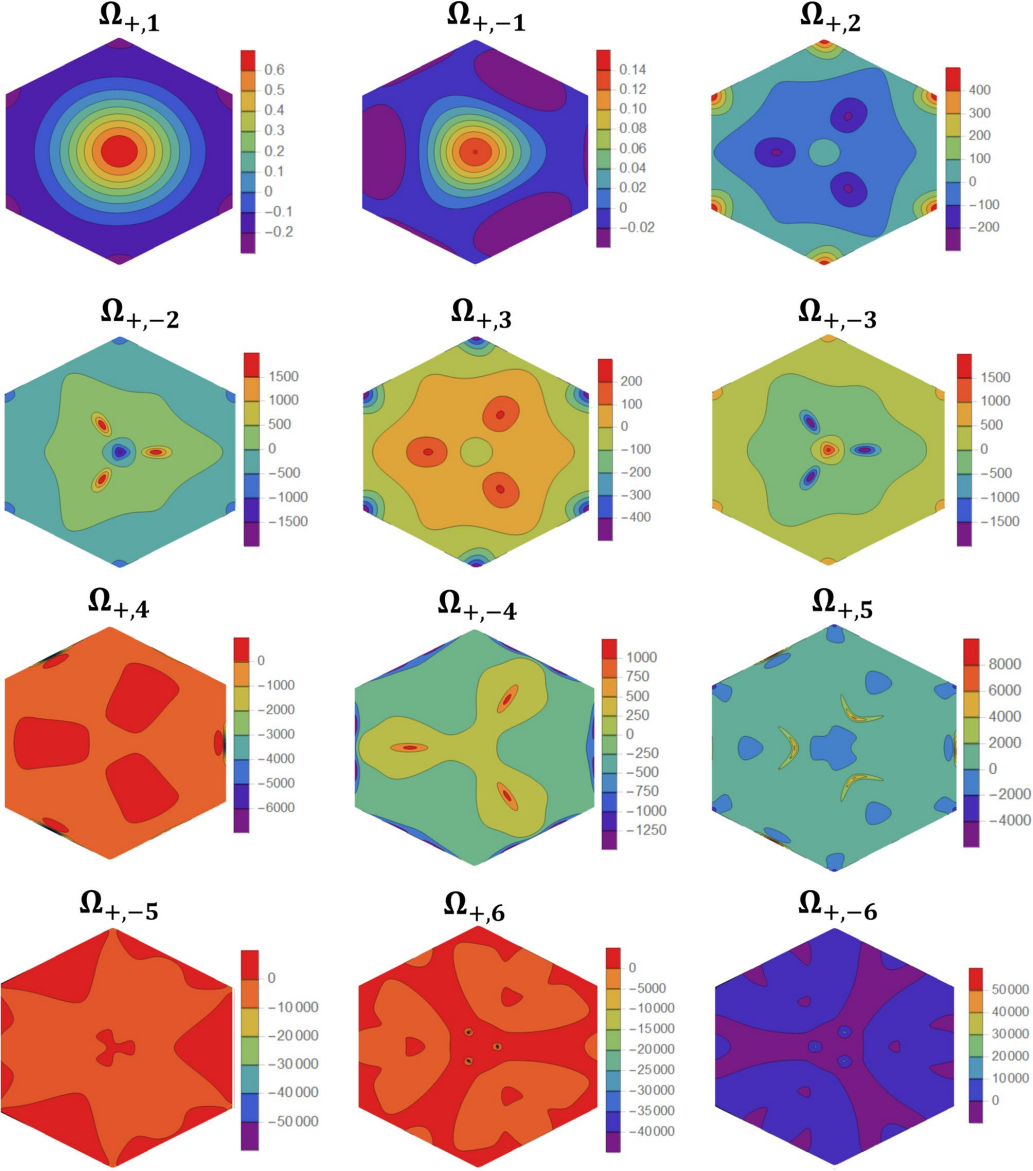
Berry curvatures plotted in the moiré BZ for selected $K -$ valley bands in a tFBL with $\theta = 3^\circ$ and $D = 0.1J$. Figures generated using Mathematica Software version 12 (free trial) https://www.wolfram.com/mathematica/.

As a sample of our numerical results, we present in Fig. 4 the $K -$ valley Berry curvatures for 12 bands ($\epsilon_{ + , \pm i}$, $i = 1, \ldots ,6$) in a tFBL with $\theta = 3^\circ$ and $D = 0.1J$. The Berry curvatures, plotted in the moiré BZ, are peaked at avoided crossings between neighboring bands. Due to the tiny band gaps, the Berry curvatures display large values which can compensate the reduced moiré BZ area and yield non-zero integer valley Chern numbers. In this context, our extensive numerical investigation demonstrate that tFBL with DMI is topologically rich, presenting multiple topological magnon bands with nonzero valley Chern numbers $C_{\mu ,i}$. Although not formally proved, we believe topological bands exist at any twist angle within the scope of the continuum approach ($\theta \le 10^\circ$). Moreover, varying the twist angle is not likely to induce an adiabatic deformation to the Berry curvatures (even the BZ changes with $\theta$) and valley Chern numbers are expected to vary with $\theta$. This is numerically confirmed and a sample of the numerically calculated $+ K -$ valley Chern numbers is presented in Table 1, for selected bands, DMI and $\theta$. With the valley Berry curvatures in hand, the valley Chern numbers are determined through numerical integration over the moiré BZ (mBZ),

$$C_{\mu ,i} = \frac{1}{2\pi }\mathop {\iint }\limits_{mBZ}^{{}} {\Omega }_{\mu ,i} \left( {\vec{q}} \right)dq_{x} dq_{y}$$

**Table 1:** $+ K$ valley Chern numbers for selected bands, $\theta$, and $D$. In addition, $C_{ + , \pm 1} = C_{ + , \pm 2} = 0$ for these choices of $D$ and $\theta$.

Chern number $\left( {{\varvec{C}}_{{{\varvec{\mu}},{\varvec{i}}}} } \right)$	${\varvec{D}} = 0.02\user2{ J}$				${\varvec{D}} = 0.1\user2{ J}$			
	$\theta = 4^\circ$	$\theta = 3.5^\circ$	$\theta = 3^\circ$	$\theta = 2.5^\circ$	$\theta = 4^\circ$	$\theta = 3.5^\circ$	$\theta = 3^\circ$	$\theta = 2.5^\circ$
${\varvec{C}}_{ + ,3}$	3	0	0	0	0	0	0	0
${\varvec{C}}_{ + , - 3}$	− 3	− 3	− 3	0	0	0	0	0
${\varvec{C}}_{ + ,4}$	1	4	4	4	− 2	− 2	− 2	− 2
${\varvec{C}}_{ + , - 4}$	− 1	− 1	− 2	− 4	− 1	− 1	− 1	− 1
${\varvec{C}}_{ + ,5}$	− 1	− 1	− 1	1	4	4	4	4
${\varvec{C}}_{ + , - 5}$	− 1	− 1	− 1	− 1	− 3	− 3	− 2	− 1
${\varvec{C}}_{ + ,6}$	3	3	3	1	4	1	− 2	− 2
${\varvec{C}}_{ + , - 6}$	− 1	− 1	− 1	− 1	− 2	1	4	2

### Tunable valley Hall and Nernst conductivities

**Figure 5:**
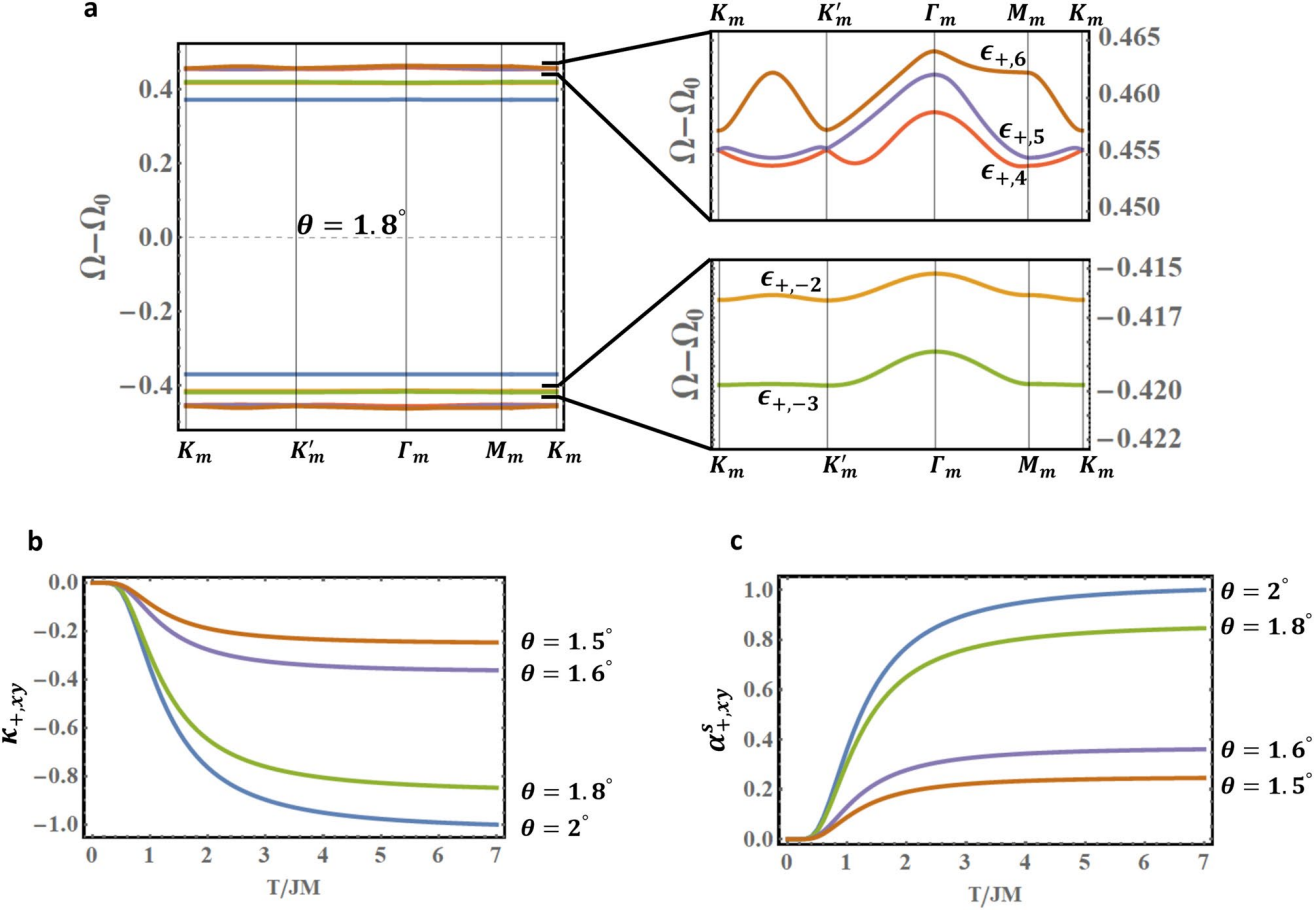
(**a**) Flat bands bundle in tFBL with $D = 0.1J$ and $\theta = 1.8^\circ$. (**b**) and (**c**) show the tunable magnon valley Hall and Nernst conductivities, induced by the topological flat bands bundle, in tFBL with $D = 0.1J$. Figures generated using Mathematica Software version 12 (free trial) https://www.wolfram.com/mathematica/.

The non-trivial valley Berry curvatures and topological bands, consequences of the DMI, induce valley thermal magnon Hall and Nernst effects in tFBL. These effects exist in tFBL at any twist angle. Of particular interest, however, are the valley Hall and Nernst conductivities induces by the topological flat bands bundle. We choose tFBL with $D = 0.1J$ characterized by a bundle of (nearly) flat bands below $2^\circ$. Figure 5a shows the first 12 flat bands $\epsilon_{ + , \pm i}$, $i = 1, \ldots ,6$ for $\theta = 1.8^\circ$. The right panel illustrates the tiny gaps between these nearly flat bands. The nonzero valley Chern numbers are investigated in Table 2 for selected angles in the range $1.5^\circ \le \theta \le 2^\circ$. The table illustrates the strong and sensitive dependence of the valley Chern numbers on $\theta$. This naturally implies significant dependence of the valley Hall and Nernst conductivities on the twist angle.

**Table 2:** Illustrates the sensitivity of flat bands’ valley Chern numbers to the twist angle in tFBL with $D = 0.1 J$.

Chern number $\left( {{\varvec{C}}_{{{\varvec{\mu}},{\varvec{i}}}} } \right)$	$\theta = 2^\circ$	$\theta = 1.8^\circ$	$\theta = 1.6^\circ$	$\theta = 1.5^\circ$
${\varvec{C}}_{ + , - 4}$	0	0	0	0
${\varvec{C}}_{ + ,5}$	2	2	0	0
${\varvec{C}}_{ + , - 5}$	− 2	− 2	− 2	− 2
${\varvec{C}}_{ + ,6}$	− 2	− 2	0	0
${\varvec{C}}_{ + , - 6}$	2	2	2	2

The $+ K$ valley Hall and Nernst conductivities, $\kappa_{ + ,xy}$ and $\alpha_{ + ,xy}^{s}$ respectively, are calculated using the standard equations ^3,7,48–51^,

$$\kappa_{ + ,xy} = - \frac{{k_{B}^{2} T}}{\hbar V}\mathop \sum \limits_{{\vec{q},i}} c_{2} \left( {g\left( {\epsilon_{ + ,i} \left( {\vec{q}} \right)} \right)} \right){\Omega }_{ + ,i} \left( {\vec{q}} \right)$$

$$\alpha_{ + ,xy}^{s} = \frac{{k_{B} }}{V}\mathop \sum \limits_{{\vec{q},i}} c_{1} \left( {g\left( {\epsilon _{ + ,i} \left( {\vec{q}} \right)} \right)} \right){\Omega }_{ + ,i} \left( {\vec{q}} \right)$$

Here $g\left( {\epsilon_{ + ,i} } \right) = \left[ {e^{{\epsilon_{ + ,i} /k_{B} T}} - 1} \right]^{ - 1}$ is the Bose–Einstein distribution function, while $c_{1} \left( x \right) = \left( {1 + x} \right)\ln \left( {1 + x} \right) - x\ln x$, and $c_{2} \left( x \right) = \left( {1 + x} \right)\left[ {\ln \left( {\frac{1 + x}{x}} \right)} \right]^{2} - \left( {\ln x} \right)^{2} - 2{\text{Li}}_{2} \left( { - x} \right)$. The symbol ${\text{Li}}_{2}$ stands for the dilogarithm function.

Figure 5b,c present the tunable valley Hall and Nernst conductivities, plotted as a function of temperature. For the selected values of the DMI and twist angles, the conductivities show a standard profile: they vanish at $T = 0$ (no thermal excitations), change exponentially for larger temperatures, and approach a constant value at elevated temperatures. The figures also illustrate the significant impact of the twist angle on the conductivities. Changing $\theta$ simultaneously affects the energies and the Berry curvatures of the bands, and eventually modifies the valley Hall and Nernst conductivities. The impact on the energy is well determined: the energy bands are gradually compressed closer to ${\Omega }_{0}$ for smaller $\theta$. The variation of the valley Chern numbers with $\theta$, however, can lead to an unsteady behavior in the valley conductivities, even when $\theta$ is varied smoothly. This is manifested in Fig.5b,c. The conductivities change slightly from $\theta = 1.5^\circ$ to $1.6^\circ$ and from $\theta = 1.8^\circ$ to $2^\circ$ since the valley Chern numbers are the same for the initial and final angles (see Table 2). This is not the case for a transition from $\theta = 1.6^\circ$ to $1.8^\circ$, where the change in the valley Chern numbers is manifested in an abrupt jump in the conductivities. A similar behavior is observed for the $- K$ valley conductivities. We note that the standard Hall and Nernst conductivities can be determined as the sum of $\pm K$ valley conductivities.

We conclude this section with a remark on valley Chern numbers and conductivities. Similar to tBLG, we expect the $\pm K$ valleys to be decoupled in tFBL which allows us to analyze their magnon excitations separately. From a theoretical point of view, it is hence possible to excite valley-polarized magnons with measurable valley conductivities, in analogy with several 2D electronic materials ^52–56^. Nevertheless, the final word should come from future experimental studies, which can hopefully realize the device and measure its topological response.

## Discussions

The present work proposes tFBL as a promising magnonic analogue for tBLG. In particular, we have focused on the topological and flat band physics induced by the DMI in tFBL.

The spin–orbit coupling is negligibly weak in tBLG. Nevertheless, magic angle flat bands in tBLG are topologically nontrivial^57–60^, interpreted in terms of the pseudo magnetic fields generated by the moiré potential^60^. Quantum anomalous Hall effect was observed in magic angle tBLG on hexagonal Boron Nitride (hBN) substrate^61–63^. The Hall effect in tBLG, however, is present only at the magic angle and cannot be tuned through the twist angle.

Similar to tBLG, the twist angle in tFBL turns into a knob that can tune the magnon spectrum and consequently the magnetic properties of tFBL. Dzyaloshinskii-Moriya (DM) spin–orbit interaction, however, is present in 2D and quasi-2D magnets with broken inversion symmetry^5,7,10,12,16^. The DMI in tFBL induces topologically rich magnon bands for any twist angle. As a result, the DMI yields topological magnon valley Hall and Nernst conductivities that can be tuned via the twist angle. Unlike tBLG, tFBL with DMI presents a continuum of magic angles which might facilitate the experimental investigation of magnonic flat bands.

Flat bands in tBLG were successfully interpreted by mapping their electronic wavefunctions to those of the lowest Landau level^64^. In our work, magnon flat bands and their topology are presented as numerical facts since the formal investigation of their origin is beyond our current scope. Another interesting topic for future investigation is the possible interesting consequences of magnon-magnon and magnon-phonon interactions. These interactions might be significant due to the band flatness.

Engineering magnon band gaps, flat bands, valley Nernst and Hall conductivities constitutes a difficult challenge for material science research. The ability to control all these characteristics via the twist angle in tFBL is indeed remarkable, and shall motivate interest in tFBL and other collinear twisted magnets. Research on 2D moiré magnets is indeed active, with a current focus on skyrmion formation and control in twisted magnetic layers with antiferromagnetic interlayer coupling^65–67^. We hope the present work opens new opportunities in the newly born field of 2D twisted magnets.

## Supplementary information

Supplementary file1

## Data Availability

The data that support the findings of the current study are available from the corresponding author upon reasonable request.
