# Supplementary material for: Magnon magic angles and tunable Hall conductivity in 2D twisted ferromagnetic bilayers
Source: Sci Rep. 2020 Sep 15;10:15069. doi: 10.1038/s41598-020-72000-y (PMC7492363; doi:10.1038/s41598-020-72000-y)
Supplement: Supplementary file 1 — Supplementary file1 [file 41598_2020_72000_MOESM1_ESM.pdf]

Supplementary Information

Magnon magic angles and tunable Hall conductivity in 2D twisted ferromagnetic bilayers

D. Ghader

Supplementary Note 1. Landau-Lifshitz equations for honeycomb ferromagnetic monolayer.

Defining the vectors $\vec{S}_D^{A/B} = S_y^{A/B}\,\hat{x} - S_x^{A/B}\,\hat{y}$, we can re-write $\mathcal{H}_{ML}$ (see expression in the main text) as

$$\mathcal{H}_{ML} = -J \sum_{\vec{R}_A,\vec{\delta}_i^A} \vec{S}^A(\vec{R}_A,t).\vec{S}^B(\vec{R}_A+\vec{\delta}_i^A,t)$$
$$+\sum_{\vec{R}_A,\vec{\gamma}_j} D_z(\vec{R}_A,\vec{R}_A+\vec{\gamma}_j)\vec{S}^A(\vec{R}_A,t).\vec{S}_D^A(\vec{R}_A+\vec{\gamma}_j,t)$$
$$+\sum_{\vec{R}_B,\vec{\gamma}_j} D_z(\vec{R}_B,\vec{R}_B+\vec{\gamma}_j)\vec{S}^B(\vec{R}_B,t).\vec{S}_D^B(\vec{R}_B+\vec{\gamma}_j,t)$$

The current form of $\mathcal{H}_{ML}$ yields the effective fields $\vec{H}^{A/B}$ on A/B sites in terms of the magnetizations $\vec{M}^{B/A}$, namely

$$\vec{H}^{A/B}(\vec{R}_{A/B},t) = -J\sum_{\vec{\delta}_i^{A/B}} \vec{M}^{B/A}(\vec{R}_{A/B}+\vec{\delta}_i^{A/B},t) + \sum_{\vec{\gamma}_j} D_z(\vec{R}_{A/B},\vec{R}_{A/B}+\vec{\gamma}_j)\vec{M}_D^{A/B}(\vec{R}_{A/B}+\vec{\gamma}_j,t)$$

Next, substituting in the Landau-Lifshitz (LL) equation of motion for the $A-$sublattice, $\partial_t\vec{M}^A = \vec{M}^A\times\vec{H}^A$, and keeping only linear terms implies

$$\partial_t M_x^A(\vec{R}_A, t) = -3JM_z M_y^A(\vec{R}_A, t) + JM_z \sum_{\vec{\delta}_i^A} M_y^B(\vec{R}_A + \vec{\delta}_i^A, t)$$

$$+M_z \sum_{\vec{\gamma}_j} D_z(\vec{R}_A, \vec{R}_A + \vec{\gamma}_j) M_x^A(\vec{R}_A + \vec{\gamma}_j, t)$$

$$\partial_t M_y^A(\vec{R}_A, t) = 3JM_z M_x^A(\vec{R}_A, t) - JM_z \sum_{\vec{\delta}_i^A} M_x^B(\vec{R}_A + \vec{\delta}_i^A, t)$$

$$+M_z \sum_{\vec{\gamma}_j} D_z(\vec{R}_A, \vec{R}_A + \vec{\gamma}_j) M_y^A(\vec{R}_A + \vec{\gamma}_j, t)$$

M_z denotes the constant z component of the magnetization. To proceed, plane wave solutions are assumed for $\vec{M}^{B/A}(\vec{r}, t)$,

$$\vec{M}^{A/B}(\vec{r}, t) = \vec{M}_{\parallel}^{A/B}(\vec{r}, t) + M_z\hat{z} = \left[M_x^{A/B}(\vec{k})\hat{x} + M_y^{A/B}(\vec{k})\hat{y}\right] e^{i(wt+\vec{k}.\vec{r})} + M_z\hat{z}$$

with frequency ω. Substituting in the time dependent LL equations, we arrive at the momentum-space equations of motion

$$i\omega M_x^A(\vec{k}) = -3JM_z M_y^A(\vec{k}) + JM_z f(\vec{k}) M_y^B(\vec{k}) + DM_z f_D(\vec{k}) M_x^A(\vec{k})$$

$$i\omega M_y^A(\vec{k}) = 3JM_z M_x^A(\vec{k}) - JM_z f(\vec{k}) M_x^B(\vec{k}) + DM_z f_D(\vec{k}) M_y^A(\vec{k})$$

with

$$f(\vec{k}) = e^{ik_y\frac{a}{\sqrt{3}}} + 2e^{-i\frac{\sqrt{3}a}{6}k_y}\cos\left(\frac{a}{2}k_x\right)$$

$$f_D(\vec{k}) = 4\, i\, sin\left(\frac{a}{2}k_x\right)\cos\left(\frac{\sqrt{3}a}{2}\, k_y\right) - 2\, i\, sin(k_x a)$$

Multiply the first equation of motion by $-i$ and summing it with the second equation implies

$$\omega M^A = [3JM_z - iDM_z f_D]M^A - JM_z f M^B$$

In a similar way, the LL equation for the B-sublattice yields

$$\omega M^B = -JM_z f^* M^A + [3JM_z + iDM_z f_D]M^B$$

We hence arrive at the momentum-space Hamiltonian for the ferromagnetic monolayer as

$$\mathcal{H}_{ML}(\vec{k}) = JM_z \begin{pmatrix} 3 - \frac{i\,D}{J} f_D(\vec{k}) & -f(\vec{k}) \\ -f^*(\vec{k}) & 3 + \frac{i\,D}{J} f_D(\vec{k}) \end{pmatrix}.$$

$\mathcal{H}_{ML}(\vec{k})$ admits 2 eigenvalues, namely

$$\Omega_\pm(\vec{k}) = \frac{\omega_\pm(\vec{k})}{JM_z} = 3 \pm \sqrt{\left|f(\vec{k})\right|^2 - \left(\frac{D}{J} f_D(\vec{k})\right)^2}$$

$\Omega_\pm(\vec{k})$ correspond to the conduction-like and valence-like bands for magnons in the 2D honeycomb ferromagnetic monolayer.

Supplementary Note 2. The Heisenberg Hamiltonian for tFBL in a less compact form.

$$
\begin{aligned}
\mathcal{H}_T = &-J \sum_{\vec{R}_{A_1},\vec{\delta}_i^A} \vec{S}^{A_1}(\vec{R}_{A_1},t).\vec{S}^{B_1}(\vec{R}_{A_1}+\vec{\delta}_i^A,t) - J \sum_{\vec{R}_{A_2},\vec{\delta}_i^A} \vec{S}^{A_2}(\vec{R}_{A_2},t).\vec{S}^{B_2}(\vec{R}_{A_2}+\vec{\delta}_i^A,t) \\
&+ \sum_{\vec{R}_{A_1},\vec{\gamma}_j} \vec{D}(\vec{R}_{A_1},\vec{R}_{A_1}+\vec{\gamma}_j).\left[\vec{S}^{A_1}(\vec{R}_{A_1},t)\times \vec{S}^{A_1}(\vec{R}_{A_1}+\vec{\gamma}_j,t)\right] \\
&+ \sum_{\vec{R}_{B_1},\vec{\gamma}_j} \vec{D}(\vec{R}_{B_1},\vec{R}_{B_1}+\vec{\gamma}_j).\left[\vec{S}^{B_1}(\vec{R}_{B_1},t)\times \vec{S}^{B_1}(\vec{R}_{B_1}+\vec{\gamma}_j,t)\right] \\
&+ \sum_{\vec{R}_{A_2},\vec{\gamma}_j} \vec{D}(\vec{R}_{A_2},\vec{R}_{A_2}+\vec{\gamma}_j).\left[\vec{S}^{A_2}(\vec{R}_{A_2},t)\times \vec{S}^{A_2}(\vec{R}_{A_2}+\vec{\gamma}_j,t)\right] \\
&+ \sum_{\vec{R}_{B_2},\vec{\gamma}_j} \vec{D}(\vec{R}_{B_2},\vec{R}_{B_2}+\vec{\gamma}_j).\left[\vec{S}^{B_2}(\vec{R}_{B_2},t)\times \vec{S}^{B_2}(\vec{R}_{B_2}+\vec{\gamma}_j,t)\right] \\
&- \sum_{\vec{R}_{A_1},\vec{R}_{A_2}} J_\perp(\vec{R}_{A_1},\vec{R}_{A_2})\,\vec{S}^{A_1}(\vec{R}_{A_1},t).\vec{S}^{A_2}(\vec{R}_{A_2},t) - \sum_{\vec{R}_{A_1},\vec{R}_{B_2}} J_\perp(\vec{R}_{A_1},\vec{R}_{B_2})\,\vec{S}^{A_1}(\vec{R}_{A_1},t).\vec{S}^{B_2}(\vec{R}_{B_2},t) \\
&- \sum_{\vec{R}_{B_1},\vec{R}_{A_2}} J_\perp(\vec{R}_{B_1},\vec{R}_{A_2})\,\vec{S}^{B_1}(\vec{R}_{B_1},t).\vec{S}^{A_2}(\vec{R}_{A_2},t) - \sum_{\vec{R}_{B_1},\vec{R}_{B_2}} J_\perp(\vec{R}_{B_1},\vec{R}_{B_2})\,\vec{S}^{B_1}(\vec{R}_{B_1},t).\vec{S}^{B_2}(\vec{R}_{B_2},t)
\end{aligned}
$$

Supplementary Note 3. Landau-Lifshitz equations in tFBL.

In the monolayer, the lattice basis vectors are $\vec{a}_1 = a\left(\frac{1}{2},\frac{\sqrt{3}}{2}\right)$ and $\vec{a}_2 = a\left(-1/2,\sqrt{3}/2\right)$ whereas the basis vectors in momentum-space are $\vec{b}_1 = \frac{2\pi}{3d}\left(\sqrt{3},1\right)$ and $\vec{b}_2 = \frac{2\pi}{3d}\left(-\sqrt{3},1\right)$. These can be generalized to the tFBL as $\vec{a}_{l,\alpha}$ and $\vec{b}_{l,\alpha}$ respectively ($\alpha = A$ or B and $l = 1$ or 2). They can be expressed as

$$\vec{a}_{2,\alpha} = R_{\theta/2}(\vec{a}_\alpha + \vec{\tau}_0)$$

$$\vec{a}_{1,\alpha} = R_{-\theta/2}\,\vec{a}_\alpha$$

$$\vec{b}_{2,\alpha} = R_{\theta/2}\,\vec{b}_\alpha$$

$$\vec{b}_{1,\alpha} = R_{-\theta/2}\,\vec{b}_\alpha$$

R_θ is a 2D anticlockwise rotation by θ. Next, the positions of the atoms on the four sublattices can be generated by the vectors

$$\vec{R}_{A_1} = \vec{R}_1 + \vec{\tau}_{1,A}$$

$$\vec{R}_{B_1} = \vec{R}_1 + \vec{\tau}_{1,B}$$

$$\vec{R}_{A_2} = \vec{R}_2 + \vec{\tau}_{2,A}$$

$$\vec{R}_{B_2} = \vec{R}_2 + \vec{\tau}_{2,B}$$

with $\vec{R}_l = n_1\vec{a}_{l,1} + n_2\vec{a}_{l,2}$ $(n_1, n_2 \,\epsilon\, \mathbb{Z})$, $\vec{\tau}_{1,A} = (0,0)$, $\vec{\tau}_{1,B} = R_{-\theta/2}\,(0,d)$, $\vec{\tau}_{2,A} = R_{\theta/2}\,[(0,-d) + \vec{\tau}_0]$, and $\vec{\tau}_{2,B} = R_{\theta/2}\,\vec{\tau}_0$.

The twist generates a moiré superlattice, with reciprocal basis vectors

$$\vec{b}_1^m = \vec{b}_{1,1} - \vec{b}_{2,1} = \frac{8\pi\sin(\theta/2\,)}{3d}(1,-\sqrt{3})$$

$$\vec{b}_2^m = \vec{b}_{1,2} - \vec{b}_{2,2} = \frac{8\pi\sin(\theta/2\,)}{3d}(1,\sqrt{3})$$

We recall the expression of the Heisenberg Hamiltonian $\mathcal{H}_T$ for the tFBL,

$$\mathcal{H}_T = -J\sum_{l,\vec{\delta}_i^A} \vec{S}^{A_l}(\vec{R}_{A_l},t).\vec{S}^{B_l}(\vec{R}_{A_l}+\vec{\delta}_i^A,t) - \sum_{\alpha,\beta} J_\perp(\vec{R}_{\alpha_1},\vec{R}_{\beta_2})\,\vec{S}^{\alpha_1}(\vec{R}_{\alpha_1},t).\vec{S}^{\beta_2}(\vec{R}_{\beta_2},t)$$
$$+\sum_{\alpha,l,\vec{\gamma}_j} \vec{D}(\vec{R}_{\alpha_l},\vec{R}_{\alpha_l}+\vec{\gamma}_j).\left[\,\vec{S}^{\alpha_l}(\vec{R}_{\alpha_l},t)\times\,\vec{S}^{\alpha_l}(\vec{R}_{\alpha_l}+\vec{\gamma}_j,t)\right] \quad \text{(S1)}$$

Similar to the monolayer case, we can write the DMI term in $\mathcal{H}_T$ as a scalar product,

$$\sum_{\alpha,l,\vec{\gamma}_j} \vec{D}(\vec{R}_{\alpha_l},\vec{R}_{\alpha_l}+\vec{\gamma}_j).\left[\,\vec{S}^{\alpha_l}(\vec{R}_{\alpha_l},t)\times\,\vec{S}^{\alpha_l}(\vec{R}_{\alpha_l}+\vec{\gamma}_j,t)\right] =$$

$$\sum_{\alpha,l,\vec{\gamma}_j} D_z(\vec{R}_{\alpha_l},\vec{R}_{\alpha_l}+\vec{\gamma}_j)\vec{S}^{\alpha_l}(\vec{R}_{\alpha_l},t).\vec{S}_D^{\alpha_l}(\vec{R}_{\alpha_l}+\vec{\gamma}_j,t)$$

with $\vec{S}_D^{\alpha_l} = S_y^{\alpha_l}\,\hat{x} - S_x^{\alpha_l}\,\hat{y}$.

We can now deduce the effective exchange fields $\vec{H}^{\alpha_l}$ acting on the magnetization $\vec{M}^{\alpha_l}$

$$\vec{H}^{\alpha_l}(\vec{R}_{\alpha_l}, t) = -J\sum_{\vec{\delta}_i^{\alpha}} \vec{M}^{\bar{\alpha}_l}(\vec{R}_{\alpha_l} + \vec{\delta}_i^{\alpha}, t) + \sum_{\vec{\gamma}_j} D_z(\vec{R}_{\alpha_l}, \vec{R}_{\alpha_l} + \vec{\gamma}_j)\vec{M}_D^{\alpha_l}(\vec{R}_{\alpha_l} + \vec{\gamma}_j, t)$$

$$-\sum_{\vec{R}_{\alpha_{\bar{l}}}} J_\perp(\vec{R}_{\alpha_l}, \vec{R}_{\alpha_{\bar{l}}})\,\vec{M}^{\alpha_{\bar{l}}}(\vec{R}_{\alpha_{\bar{l}}}, t) - \sum_{\vec{R}_{\bar{\alpha}_{\bar{l}}}} J_\perp(\vec{R}_{\alpha_l}, \vec{R}_{\bar{\alpha}_{\bar{l}}})\,\vec{M}^{\bar{\alpha}_{\bar{l}}}(\vec{R}_{\bar{\alpha}_{\bar{l}}}, t)$$

(S2)

where we have used the convention that if $\alpha = A$ then $\bar{\alpha} = B$ and vice versa. Same convention assumed for l and $\bar{l}$.

We assume harmonic time dependence (with frequency ω) for the magnetizations. The x and y components of the LL equations of motion, $\partial_t \vec{M}^{\alpha_l} = \vec{M}^{\alpha_l} \times \vec{H}^{\alpha_l}$, yield 2 equations of motion for each sublattice α_l. Combining the x and y equations yield

$$\omega M^{\alpha_l}(\vec{R}_{\alpha_l}) = \left[3JM_z + M_z\sum_{\vec{R}_{\alpha_{\bar{l}}}} J_\perp(\vec{R}_{\alpha_l}, \vec{R}_{\alpha_{\bar{l}}}) + M_z\sum_{\vec{R}_{\bar{\alpha}_{\bar{l}}}} J_\perp(\vec{R}_{\alpha_l}, \vec{R}_{\bar{\alpha}_{\bar{l}}})\right] M^{\alpha_l}(\vec{R}_{\alpha_l})$$

$$-JM_z\sum_{\vec{\delta}_i^{\alpha}} M^{\bar{\alpha}_l}(\vec{R}_{\alpha_l} + \vec{\delta}_i^{\alpha}) - iM_z\sum_{\vec{\gamma}_j} D_z(\vec{R}_{\alpha_l}, \vec{R}_{\alpha_l} + \vec{\gamma}_j)M^{\alpha_l}(\vec{R}_{\alpha_l} + \vec{\gamma}_j, t)$$

$$-M_z\sum_{\vec{R}_{\alpha_{\bar{l}}}} J_\perp(\vec{R}_{\alpha_l}, \vec{R}_{\alpha_{\bar{l}}})\,M^{\alpha_{\bar{l}}}(\vec{R}_{\alpha_{\bar{l}}}) - M_z\sum_{\vec{R}_{\bar{\alpha}_{\bar{l}}}} J_\perp(\vec{R}_{\alpha_l}, \vec{R}_{\bar{\alpha}_{\bar{l}}})\,M^{\bar{\alpha}_{\bar{l}}}(\vec{R}_{\bar{\alpha}_{\bar{l}}})$$

(S3)

with $M^{\alpha_l} = M_x^{\alpha_l} + iM_y^{\alpha_l}$.

We next expand the magnetization amplitudes in terms of Bloch waves

$$\frac{\omega}{\sqrt{N_l}}\sum_{\vec{k}_l'} e^{i\vec{k}_l'.\vec{R}_{\alpha_l}} u_{\alpha_l}(\vec{k}_l') = -\frac{JM_z}{\sqrt{N_l}}\sum_{\vec{k}_l'} f_{ex}^{\alpha}(\vec{k}_l')\, e^{i\vec{k}_l'.\vec{R}_{\alpha_l}}\, u_{\bar{\alpha}_l}(\vec{k}_l') +$$

$$\frac{1}{\sqrt{N_l}}\left[3M_zJ + M_zDf_D^{\alpha}(\vec{k}_l') + M_z\sum_{\vec{R}_{\alpha_{\bar{l}}}} J_\perp(\vec{R}_{\alpha_l},\vec{R}_{\alpha_{\bar{l}}}) \; + M_z\sum_{\vec{R}_{\bar{\alpha}_{\bar{l}}}} J_\perp(\vec{R}_{\alpha_l},\vec{R}_{\bar{\alpha}_{\bar{l}}})\right]\sum_{\vec{k}_l'} e^{i\vec{k}_l'.\vec{R}_{\alpha_l}} u_{\alpha_l}(\vec{k}_l')$$

$$-\frac{M_z}{\sqrt{N_{\bar{l}}}}\sum_{\vec{R}_{\alpha_{\bar{l}}},\vec{k}_{\bar{l}}} J_\perp(\vec{R}_{\alpha_l},\vec{R}_{\alpha_{\bar{l}}}) e^{i\vec{k}_{\bar{l}}.\vec{R}_{\alpha_{\bar{l}}}} u_{\alpha_{\bar{l}}}(\vec{k}_{\bar{l}}) \; -\frac{M_z}{\sqrt{N_{\bar{l}}}}\sum_{\vec{R}_{\bar{\alpha}_{\bar{l}}},\vec{k}_{\bar{l}}} J_\perp(\vec{R}_{\alpha_l},\vec{R}_{\bar{\alpha}_{\bar{l}}}) e^{i\vec{k}_{\bar{l}}.\vec{R}_{\bar{\alpha}_{\bar{l}}}} u_{\bar{\alpha}_{\bar{l}}}(\vec{k}_{\bar{l}}) \tag{S4}$$

N_l and $N_{\bar{l}}$ are the number of unit cells while $\vec{k}_l'$ and $\vec{k}_{\bar{l}}$ are wave vectors in layers l and $\bar{l}$. We have also defined

$$f_{ex}^{A}(\vec{k}_l') = \sum_{\vec{\delta}_i^A} e^{i\vec{k}_l'.\vec{\delta}_i^A} = e^{ik_{l,y}'\frac{a}{\sqrt{3}}} + 2e^{-i\frac{\sqrt{3}a}{6}k_{l,y}'}\cos\left(\frac{a}{2}k_{l,x}'\right) = \left(f_{ex}^{B}(\vec{k}_l')\right)^*$$

$$f_D^{A}(\vec{k}_l') = \sum_{\vec{\gamma}_j} e^{i\vec{k}_l'.\vec{\gamma}_j} = 4sin\left(\frac{a}{2}k_x\right)\cos\left(\frac{\sqrt{3}a}{2}k_y\right) - 2sin(k_xa) = -f_D^{B}(\vec{k}_l') \tag{S5}$$

Finally, we multiply equation S4 by $e^{-i\vec{k}_l.\vec{R}_{\alpha_l}}$ and sum the whole equation over $\vec{R}_{\alpha_l}$ to get

$$\omega\, u_{\alpha_l}(\vec{k}_l) = [3M_zJ + M_zDf_D^{\alpha}(\vec{k}_l')]u_{\alpha_l}(\vec{k}_l) - JM_zf_{ex}^{\alpha}(\vec{k}_l')u_{\bar{\alpha}_l}(\vec{k}_l)$$

$$+M_z\sum_{\vec{k}_l'}\left[\mathcal{J}^{\alpha_l,\alpha_{\bar{l}}}(\vec{k}_l,\vec{k}_l') + \mathcal{J}^{\alpha_l,\bar{\alpha}_{\bar{l}}}(\vec{k}_l,\vec{k}_l')\right] u_{\alpha_l}(\vec{k}_l')$$

$$-M_z\sum_{\vec{k}_{\bar{l}}}\mathcal{J}_\perp^{\alpha_l,\alpha_{\bar{l}}}(\vec{k}_l,\vec{k}_{\bar{l}})\, u_{\alpha_{\bar{l}}}(\vec{k}_{\bar{l}}) - M_z\sum_{\vec{k}_{\bar{l}}}\mathcal{J}_\perp^{\alpha_l,\bar{\alpha}_{\bar{l}}}(\vec{k}_l,\vec{k}_{\bar{l}})\, u_{\bar{\alpha}_{\bar{l}}}(\vec{k}_{\bar{l}}) \tag{S6}$$

with the interlayer coefficients defined as

$$\mathcal{J}_\perp^{\alpha_l,\alpha_{\bar{l}}}(\vec{k}_l,\vec{k}_{\bar{l}}) = \frac{1}{\sqrt{N_lN_{\bar{l}}}}\sum_{\vec{R}_{\alpha_l},\vec{R}_{\alpha_{\bar{l}}}} e^{-i\vec{k}_l.\vec{R}_{\alpha_l}} J_\perp(\vec{R}_{\alpha_l},\vec{R}_{\alpha_{\bar{l}}}) e^{i\vec{k}_{\bar{l}}.\vec{R}_{\alpha_{\bar{l}}}} \tag{S7a}$$

$$\mathcal{J}_{\perp}^{\alpha_l,\bar{\alpha}_{\bar{l}}}\left(\vec{k}_l,\vec{k}_{\bar{l}}\right)=\frac{1}{\sqrt{N_l N_{\bar{l}}}}\sum_{\vec{R}_{\alpha_l},\vec{R}_{\bar{\alpha}_{\bar{l}}}} e^{-i\vec{k}_l.\vec{R}_{\alpha_l}} J_{\perp}\left(\vec{R}_{\alpha_l},\vec{R}_{\bar{\alpha}_{\bar{l}}}\right) e^{i\vec{k}_{\bar{l}}.\vec{R}_{\bar{\alpha}_{\bar{l}}}} \tag{S7b}$$

while the intralayer coefficients read

$$\mathcal{J}^{\alpha_l,\alpha_{\bar{l}}}\left(\vec{k}_l,\vec{k}_l'\right)=\frac{1}{N_l}\sum_{\vec{R}_{\alpha_l},\vec{R}_{\alpha_{\bar{l}}}} e^{-i\left(\vec{k}_l-\vec{k}_l'\right).\vec{R}_{\alpha_l}} J_{\perp}\left(\vec{R}_{\alpha_l},\vec{R}_{\alpha_{\bar{l}}}\right) \tag{S8a}$$

$$\mathcal{J}^{\alpha_l,\bar{\alpha}_{\bar{l}}}\left(\vec{k}_l,\vec{k}_l'\right)=\frac{1}{N_l}\sum_{\vec{R}_{\alpha_l},\vec{R}_{\bar{\alpha}_{\bar{l}}}} e^{-i\left(\vec{k}_l-\vec{k}_l'\right).\vec{R}_{\alpha_l}} J_{\perp}\left(\vec{R}_{\alpha_l},\vec{R}_{\bar{\alpha}_{\bar{l}}}\right) \tag{S8b}$$

The interlayer terms in the LL equations are qualitatively identical to those encountered in the electronic theory of tBLG. The Bistritzer - MacDonald continuum approach yields the identities

$$\begin{aligned}\mathcal{J}_{\perp}^{\alpha_l,\alpha_{\bar{l}}}\left(\vec{K}_l+\vec{q}_l,\vec{K}_{\bar{l}}+\vec{q}_{\bar{l}}\right)=\frac{J_{\perp}}{3}\Big[&\delta_{\vec{q}_l-\vec{q}_{\bar{l}},-(\vec{K}_l-\vec{K}_{\bar{l}})}+e^{i\vec{b}_{l,2}.\vec{\tau}_{l,\alpha}}\ e^{-i\vec{b}_{\bar{l},2}.\vec{\tau}_{\bar{l},\alpha}}\ \delta_{\vec{q}_l-\vec{q}_{\bar{l}},-(\vec{K}_l-\vec{K}_{\bar{l}}+\vec{b}_{l,2}-\vec{b}_{\bar{l},2})}\\ &+e^{-i\vec{b}_{l,1}.\vec{\tau}_{l,\alpha}}\ e^{i\vec{b}_{\bar{l},1}.\vec{\tau}_{\bar{l},\alpha}}\ \delta_{\vec{q}_l-\vec{q}_{\bar{l}},-(\vec{K}_l-\vec{K}_{\bar{l}}-\vec{b}_{l,1}+\vec{b}_{\bar{l},1})}\Big]\end{aligned} \tag{S9a}$$

$$\begin{aligned}\mathcal{J}_{\perp}^{\alpha_l,\bar{\alpha}_{\bar{l}}}\left(\vec{K}_l+\vec{q}_l,\vec{K}_{\bar{l}}+\vec{q}_{\bar{l}}\right)=\frac{J_{\perp}}{3}\Big[&\delta_{\vec{q}_l-\vec{q}_{\bar{l}},-(\vec{K}_l-\vec{K}_{\bar{l}})}+e^{i\vec{b}_{l,2}.\vec{\tau}_{l,\alpha}}\ e^{-i\vec{b}_{\bar{l},2}.\vec{\tau}_{\bar{l},\bar{\alpha}}}\ \delta_{\vec{q}_l-\vec{q}_{\bar{l}},-(\vec{K}_l-\vec{K}_{\bar{l}}+\vec{b}_{l,2}-\vec{b}_{\bar{l},2})}\\ &+e^{-i\vec{b}_{l,1}.\vec{\tau}_{l,\alpha}}\ e^{i\vec{b}_{\bar{l},1}.\vec{\tau}_{\bar{l},\bar{\alpha}}}\ \delta_{\vec{q}_l-\vec{q}_{\bar{l}},-(\vec{K}_l-\vec{K}_{\bar{l}}-\vec{b}_{l,1}+\vec{b}_{\bar{l},1})}\Big]\end{aligned} \tag{S9b}$$

We now consider the intralayer coefficients presented in equations S8a and S8b, absent in the electronic theory of graphene. The starting point is the Fourier transform of $J_{\perp}\left(\vec{R}_{\alpha_l},\vec{R}_{\alpha_{\bar{l}}}\right)$,

$$\mathcal{J}^{\alpha_l,\alpha_{\bar{l}}}(\vec{k}_l,\vec{k}'_l) = \frac{1}{N_l}\sum_{\vec{R}_{\alpha_l},\vec{R}_{\alpha_{\bar{l}}}} e^{-i(\vec{k}_l-\vec{k}'_l).\vec{R}_{\alpha_l}} J_\perp(\vec{R}_{\alpha_l},\vec{R}_{\alpha_{\bar{l}}})$$

$$= \frac{1}{N_l}\int_{\mathbb{R}^2}\frac{d^2\vec{p}}{(2\pi)^2}\tilde{J}_\perp(\vec{p})\sum_{\vec{R}_l} e^{-i(\vec{k}_l-\vec{k}'_l-\vec{p}).(\vec{R}_l+\vec{\tau}_{l,\alpha})}\sum_{\vec{R}_{\bar{l}}} e^{-i\vec{p}.(\vec{R}_{\bar{l}}+\vec{\tau}_{\bar{l},\alpha})}$$

$$= N_{\bar{l}}\int_{\mathbb{R}^2}\frac{d^2\vec{p}}{(2\pi)^2}\tilde{J}_\perp(\vec{p})\sum_{\vec{G}_l,\vec{G}_{\bar{l}}} e^{-i\vec{G}_l.\vec{\tau}_{l,\alpha}}\, e^{-i\vec{G}_{\bar{l}}.\vec{\tau}_{\bar{l},\alpha}}\,\delta_{\vec{k}_l-\vec{k}'_l-\vec{p},\vec{G}_l}\,\delta_{\vec{p},\vec{G}_{\bar{l}}}$$

$$= \frac{1}{A}\sum_{\vec{G}_l,\vec{G}_{\bar{l}}}\tilde{J}_\perp(\vec{G}_{\bar{l}})e^{-i\vec{G}_l.\vec{\tau}_{l,\alpha}}\, e^{i\vec{G}_{\bar{l}}.\vec{\tau}_{\bar{l},\alpha}}\,\delta_{\vec{k}_l-\vec{k}'_l,\vec{G}_l-\vec{G}_{\bar{l}}}$$

In the present case, both $\vec{k}_l$ and $\vec{k}'_l$ are expanded near K_l,

$$\mathcal{J}^{\alpha_l,\alpha_{\bar{l}}}(\vec{K}_l+\vec{q}_l,\vec{K}_l+\vec{q}'_l) = \frac{1}{A}\sum_{\vec{G}_l,\vec{G}_{\bar{l}}}\tilde{J}_\perp(\vec{G}_{\bar{l}})e^{-i\vec{G}_l.\vec{\tau}_{l,\alpha}}\, e^{i\vec{G}_{\bar{l}}.\vec{\tau}_{\bar{l},\alpha}}\,\delta_{\vec{q}_l-\vec{q}'_l,\vec{G}_l-\vec{G}_{\bar{l}}} \tag{S10a}$$

Similarly

$$\mathcal{J}^{\alpha_l,\bar{\alpha}_{\bar{l}}}(\vec{K}_l+\vec{q}_l,\vec{K}_l+\vec{q}'_l) = \frac{1}{A}\sum_{\vec{G}_l,\vec{G}_{\bar{l}}}\tilde{J}_\perp(\vec{G}_{\bar{l}})e^{-i\vec{G}_l.\vec{\tau}_{l,\alpha}}\, e^{i\vec{G}_{\bar{l}}.\vec{\tau}_{\bar{l},\bar{\alpha}}}\,\delta_{\vec{q}_l-\vec{q}'_l,\vec{G}_l-\vec{G}_{\bar{l}}} \tag{S10b}$$

Near K_l, the vectors $\vec{q}_l-\vec{q}'_l$ in equations S10 are very small and match only moiré reciprocal lattice vectors $\vec{G}^m = \vec{G}_l-\vec{G}_{\bar{l}} = \pm(R_{-\theta/2}\,\vec{G}-R_{\theta/2}\,\vec{G})$. Here $\vec{G}=n_1\vec{b}_1+n_2\vec{b}_2$ is a reciprocal lattice vector of the unrotated honeycomb monolayer. The summation in S10 hence reduces to a summation over $\vec{G}$ of the unrotated lattice. For example,

$$\mathcal{J}^{A_1,A_2}(\vec{K}_1+\vec{q}_1,\vec{K}_1+\vec{q}'_1) = \frac{1}{A}\sum_{\vec{G}}\tilde{J}_\perp(|\vec{G}|)e^{-i\vec{G}.(0,0)}\, e^{i\vec{G}.[(0,-d)+\vec{\tau}_0]}\,\delta_{\vec{q}_1-\vec{q}'_1,R_{-\theta/2}\,\vec{G}-R_{\theta/2}\,\vec{G}} \tag{S11}$$

In the summation present in equation 11, we only need to consider the most relevant contributions, namely $\vec{G} = \vec{0}, \pm\vec{b}_1$, and $\pm\,\vec{b}_2$. Consequently,

$$\mathcal{J}^{A_1,A_2}\left(\vec{K}_1 + \vec{q}_1, \vec{K}_1 + \vec{q}_1'\right) =$$

$$\frac{\tilde{J}_\perp(0)}{A}\,\delta_{\vec{q}_1-\vec{q}_1',\vec{0}} + \frac{\tilde{J}_\perp\left(\sqrt{3}\times|\vec{K}|\right)}{A}\left[e^{i\left(\vec{b}_1.\vec{\tau}_0-\varphi\right)}\delta_{\vec{q}_1-\vec{q}_1',\vec{G}_1^m} + e^{-i\left(\vec{b}_1.\vec{\tau}_0-\varphi\right)}\delta_{\vec{q}_1-\vec{q}_1',-\vec{G}_1^m}\right]$$

$$+\frac{\tilde{J}_\perp\left(\sqrt{3}\times|\vec{K}|\right)}{A}\left[e^{i\left(\vec{b}_2.\vec{\tau}_0-\varphi\right)}\delta_{\vec{q}_1-\vec{q}_1',\vec{G}_2^m} + e^{-i\left(\vec{b}_2.\vec{\tau}_0-\varphi\right)}\delta_{\vec{q}_1-\vec{q}_1',-\vec{G}_2^m}\right]$$

with $\varphi = 2\pi/3$, $\vec{G}_1^m = R_{-\theta/2}\,\vec{b}_1 - R_{\theta/2}\,\vec{b}_1$ and $\vec{G}_2^m = R_{-\theta/2}\,\vec{b}_2 - R_{\theta/2}\,\vec{b}_2$. We have also used the fact $\tilde{J}_\perp\left(|\vec{b}_1|\right) = \tilde{J}_\perp\left(|\vec{b}_2|\right) = \tilde{J}_\perp\left(\sqrt{3}\times|\vec{K}_1|\right) = \tilde{J}_\perp\left(\sqrt{3}\times|\vec{K}|\right)$.

Before proceeding, we note that for the case $\theta = 0$, the summation in S11 becomes infinite and $\mathcal{J}^{\alpha_l,\alpha_{\bar{l}}}$ converges to $J_\perp\left(d_{\alpha_l,\alpha_{\bar{l}}}\right)$, where $d_{\alpha_l,\alpha_{\bar{l}}}$ denotes the distance between sites α_l and $\alpha_{\bar{l}}$. This perfectly reproduces the AA/AB stacking cases.

In van der Waals magnetic materials, the interlayer Fourier transform $\tilde{J}_\perp(k)$ is extremely sharp and $\tilde{J}_\perp\left(\sqrt{3}\times|\vec{K}|\right)$ is negligible compared to $\tilde{J}_\perp(0)$. We hence arrive at the simple expressions

$$\mathcal{J}^{\alpha_l,\alpha_{\bar{l}}}\left(\vec{K}_l + \vec{q}_l, \vec{K}_l + \vec{q}_l'\right) \approx \mathcal{J}^{\alpha_l,\bar{\alpha}_{\bar{l}}}\left(\vec{K}_l + \vec{q}_l, \vec{K}_l + \vec{q}_l'\right) \approx \frac{\tilde{J}_\perp(0)}{A}\,\delta_{\vec{q}_1,\vec{q}_1'} \tag{S12}$$

With this faithful approximation, the magnon theory is independent of $\vec{\tau}_0$ as in tBLG (we set $\vec{\tau}_0 = \vec{0}$). Substituting equations S9 and S12 in S6 then expanding $f_{ex}^{\alpha}\left(\vec{k}_l'\right)$ and f_D^{α} near K_l and $K_{\bar{l}}$ yields the final expressions of the LL equations (equations 3 in the main text).

Supplementary Note 4. Numerical results for magnon band reconstruction.

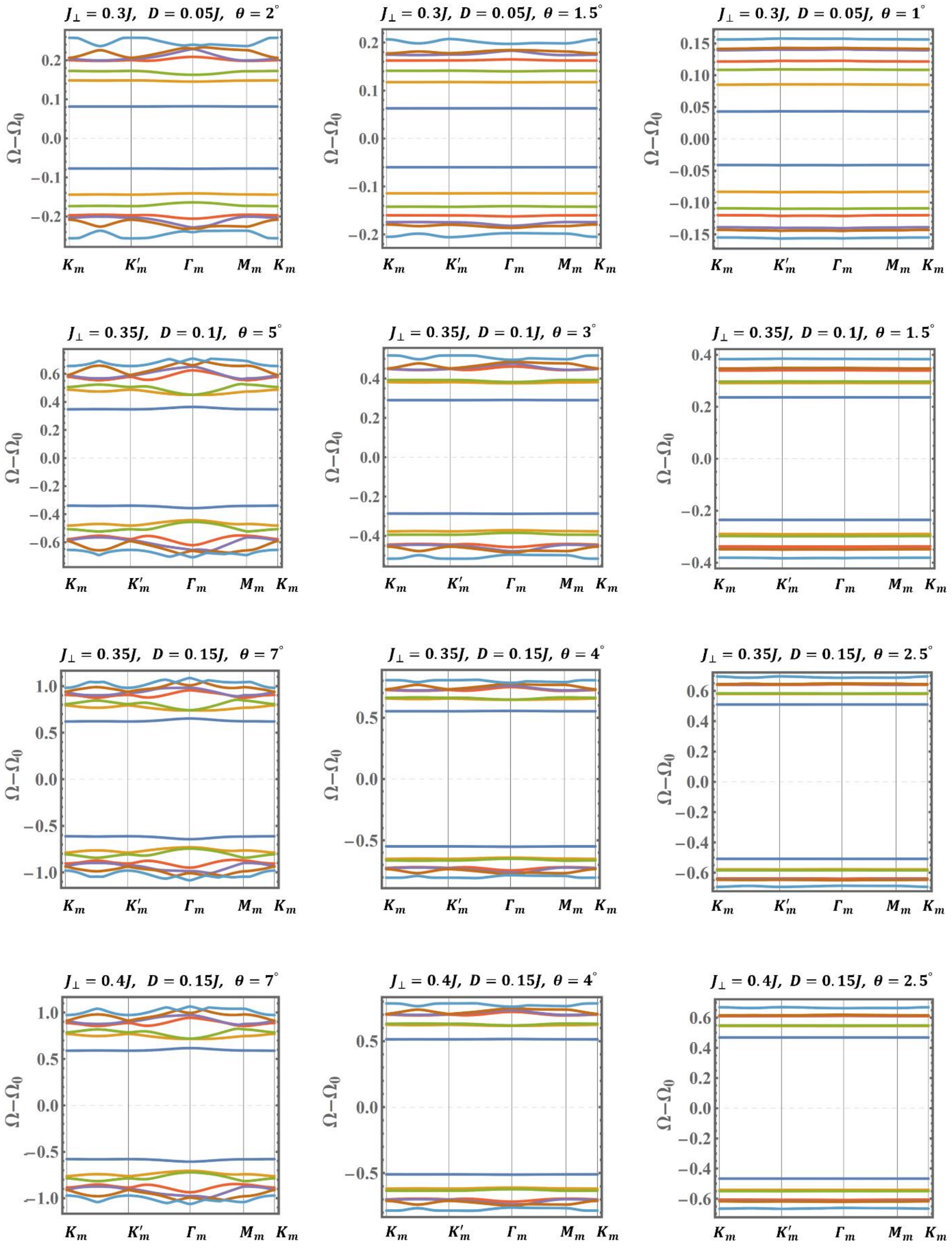

Figure S1: Reconstruction of the $K -$ valley magnon spectrum for selected values of $J_\perp$ and D.
